# S1 guideline for imaging diagnostics for skin diseases

**DOI:** 10.1111/ddg.15883

**Published:** 2025-11-16

**Authors:** Maximilian Deußing, Sandra Schuh, Janis Thamm, Deborah Winkler, Simon Schneider, Teresa Nau, Ulf Darsow, Viktor Schnabel, Martina Ulrich, Lynhda Nguyen, Denis Frenzel, Chiara Fischer, Cristel Ruini, Vasilis Ntziachristos, Martin Kaatz, Hjalmar Kurzen, Bernd Kardorff, Rudolf Herbst, Sonja Grunewald, Elke Sattler, Julia Welzel, Daniela Hartmann

**Affiliations:** ^1^ Department of Dermatology and Allergy LMU Munich Munich Germany; ^2^ Department of Dermatology Allergology and Laser Medicine Munich Municipal Hospital Munich Germany; ^3^ Department of Dermatology and Allergology University Hospital Augsburg Augsburg Germany; ^4^ Department of Dermatology and Allergology Technical University of Munich Munich Germany; ^5^ Department of Dermatology Venereology and Allergology University Hospital Leipzig Leipzig Germany; ^6^ Dermatologie am Regierungsviertel Berlin Germany; ^7^ Department of Dermatology and Venereology University Hospital Hamburg‐Eppendorf (UKE) Hamburg Germany; ^8^ Praxisklinik Starnberg Starnberg Germany; ^9^ Department of Biological Imaging Technical University of Munich Munich Germany; ^10^ Department of Dermatology Sapienza University of Rome Rome Italy; ^11^ Institute of Biological and Medical Imaging Helmholtz Center Munich Neuherberg Germany Chair of Biological Imaging Central Institute for Translational Cancer Research (TranslaTUM) School of Medicine Technical University of Munich Munich Germany; ^12^ Department of Dermatology DRK Hospital Chemnitz‐Rabenstein Chemnitz Germany; ^13^ Freising Skin and Laser Center Freising Germany; ^14^ Group Practice for Dermatology Allergology Phlebology and Laser Medicine Prof. Dr. Kardorff medermis clinics Mönchengladbach Germany; ^15^ Department of Dermatology and Allergology Helios Hospital Erfurt Erfurt Germany

**Keywords:** Basal cell carcinoma, malignant melanoma, non‐invasive imaging, physical diagnosis, squamous cell carcinoma

## Abstract

Non‐invasive imaging techniques allow a quick and easy in vivo examination of the skin with different penetration depths and resolution depending on the applied technology.

Established methods such as dermoscopy and high‐resolution sonography of the skin have been an integral part of everyday life for decades. Additionally, new emerging techniques such as optical coherence tomography (OCT), reflectance confocal microscopy (RCM), and line‐field confocal OCT (LC‐OCT) have entered clinical practice. Multiphoton tomography and optoacoustic imaging are also considered promising new methods.

RCM and LC‐OCT can also be used *ex vivo* on freshly excised tissue, for example in Moh's surgery margin assessment.

The data generated by all imaging methods is ideal for the application of AI‐based algorithms to increase diagnostic accuracy and support experienced users.

All mentioned methods have preferred indications depending on their strengths and limitations, both in skin tumor diagnostics and in inflammatory, infectious and parasitic dermatoses. The following guideline provides an overview of the various devices and techniques, explains how each method works and provides the current study situation with indications and limitations of each procedure.

## ABOUT THIS GUIDELINE

This version of the guideline is a short version of the complete guideline, which is freely available as online supplement and at www.awmf.org. A complete list of references and an overview table on which the recommendations and statements of this guideline are based, as well as the conflicts of interest of the participating authors are provided in the long version or in the guideline report.

This first German guideline on imaging diagnostics for skin diseases was developed under the guidance of the German Dermatological Society (*Deutsche Dermatologische Gesellschaft*, DDG) and the Working Group Physical Diagnostics in Dermatology (*Arbeitsgemeinschaft für Physikalische Diagnostik in der Dermatologie*, ApDD) and is based on a systematic literature search and the consensus of the expert group. The guideline includes recommendations on technique, indications, evidence, and limitations of the various imaging methods.

Given that medicine is subject to a continuous process of development, any information, in particular on diagnostic and therapeutic procedures, can only reflect the state of knowledge at the time of printing the guidelines. The utmost care was taken with respect to the stated therapy recommendations as well as selection and the dosage of drugs. Nevertheless, users are advised to refer to the package leaflet or the manufacturer's summary of product characteristics and to consult a specialist in case of doubt. The user assumes responsibility for each diagnostic and therapeutic application, medication, and dosage. In this guideline, registered trademarks (protected trademarks) are not specifically identified. The absence of a respective reference does not imply that the trade name is not protected (free of charge).

## REFLECTANCE CONFOCAL MICROSCOPY (RCM)

### Technique

Reflectance confocal microscopy (RCM) is suited particularly well for the non‐invasive diagnosis of melanocytic and epithelial skin tumors.^1^ Laser light of a selected wavelength is focused on a plane within the skin, where the light is reflected at interfaces with high refractive index (keratin, melanin, and collagen) and is then directed to a detector. Due to an upstream pinhole aperture, only signals from a predefined horizontal plane are used for imaging. While this approach allows for high‐resolution visualization of changes close to the surface with microscopic resolution of 1 to 3 µm in horizontal sections, it also limits the penetration depth into the skin.

### Instruments

For reflectance confocal microscopy, instruments with one or multiple lasers are used as light sources. They can be used for both in vivo and *ex vivo* assessment of the skin. Given that the laser energy at tissue level is lower than 30 mW, there is no danger to the tissue being examined or to the human eye (laser class I).

### Indications

#### Melanocytic lesions

Due to the high refractive index of melanin, melanocytic lesions can be visualized particularly well. Consequently, morphological image characteristics for the differentiation of benign and suspicious lesions have been established.^2^, ^3^

**FIGURE 1:**
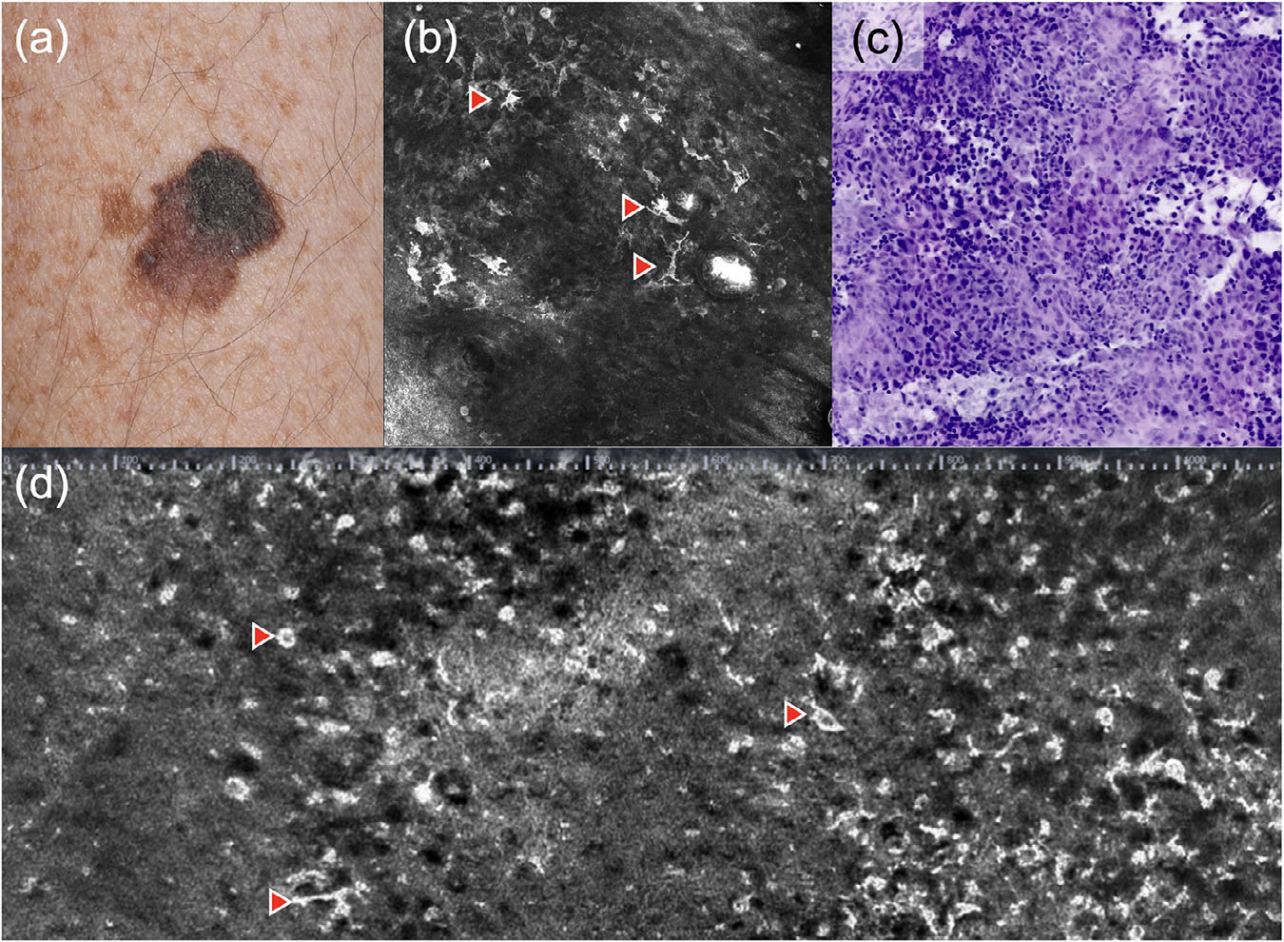
Superficial spreading melanoma in various imaging techniques. (a) Clinical image; (b) horizontal in vivo reflectance confocal microscopy (VivaScope 1500, 750 µm × 750 µm, VivaScope GmbH, Munich, Germany); (c) ex vivo reflectance confocal microscopy of a nodular melanoma (VivaScope 2500M‐G4, VivaScope GmbH, Munich, Germany) showing dermal nests of atypical melanocytes in digital H&E mode; (d) vertical line‐field confocal optical coherence tomography (DeepLive, DAMAE Medical, Paris, France) showing pagetoid and dendritic cells (arrows).

In this context, elimination of the normal epidermal architecture (atypical honeycomb pattern) and the normal structure of the dermal‐epidermal junction (DEJ, abrupt DEJ), lacking definition of dermal papillae (non‐edged papillae), presence of large, highly refractive cells with prominent nuclei in higher epidermal layers (round and dendritic pagetoid cells [Figure 1]), irregular nests of atypical melanocytes (dense and sparse nests, cerebriform nests), and small, highly refractive particles (inflammatory particles) are considered the most important criteria for malignant transformation. While diagnosis of amelanotic melanomas by RCM is more difficult, asymmetric, pigmented follicles, ≥ 3 atypical cells in five fields, and focal follicular extension of atypical cells at DEJ are considered key criteria for the differentiation from other skin tumors.^4^

Numerous studies show that RCM results in improved specificity in melanoma diagnostics compared to dermoscopy alone, especially in the case of unclear lesions. This has also been demonstrated in randomized controlled studies.^5^, ^6^, ^7^, ^8^ Ultimately, the use of RCM reduces the number of unnecessary excisions and enables early detection of even thin melanomas. Moreover, RCM can significantly reduce the *number needed to excise* (NNE),^8^, ^9^ thus reducing the costs for the healthcare system.^9^

#### Basal cell carcinoma

RCM is also suitable for the examination of nonmelanoma skin cancer.^10^, ^11^, ^12^, ^13^, ^14^, ^15^, ^16^

**FIGURE 2:**
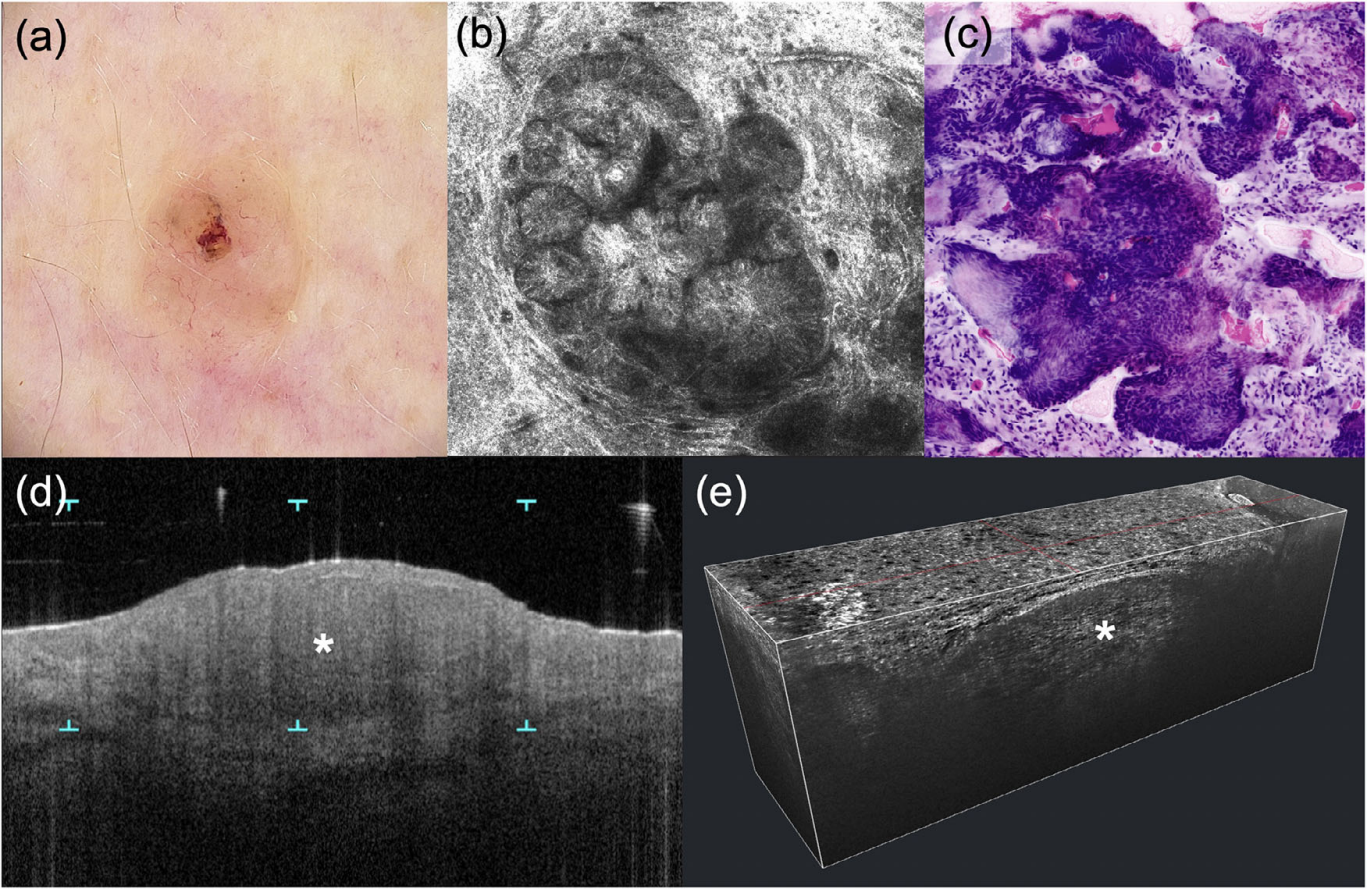
Solid basal cell carcinoma. (a) Dermoscopic view (× 20); (b) in vivo reflectance confocal microscopy (VivaScope 1500, 750 µm × 750 µm, VivaScope GmbH, Munich, Germany) showing characteristic tumor islands; (c) ex vivo reflectance confocal microscopy (VivaScope 2500M‐G4, VivaScope GmbH, Munich, Germany) in digital H&E mode showing tumor cell aggregates; (d) optical coherence tomography (VivoSight, Michelson Diagnostics Ltd, Kent, UK) in vertical plane showing the tumor nodule (star); (e) line‐field confocal optical coherence tomography (DeepLive, DAMAE Medical, Paris, France) in 3D representation.

The following five main criteria have been described for diagnosis of basal cell carcinoma: elongated, monomorphic nuclei, polarization of these cells along one axis, pronounced inflammatory infiltrate, more and dilated vessels, and loss of epidermal honeycomb structure.^17^ Islands of tumor cells with peripheral palisading in the dermis, separated from the connective tissue by a dark cleft, are also considered a characteristic feature (Figure 2). Histologically, this optical clefting corresponds to the accumulation of mucin.^18^ In a large multicenter study, a high sensitivity of RCM of 100% and a specificity of 88.5% was shown for the diagnosis of basal cell carcinoma.^18^

#### Actinic keratosis and squamous cell carcinoma

In RCM, actinic keratoses are characterized by a loss of normal honeycomb structure with atypia and pleomorphism of epidermal keratinocytes, parakeratosis, detached corneocytes in the stratum corneum, and solar elastosis, as well as blood vessel dilation.^19^, ^20^, ^21^, ^22^, ^23^

Apart from the atypical honeycomb structure, Bowen's disease/squamous cell carcinomas in situ present with dyskeratosis and, typically, glomerular vessels.^24^, ^25^

#### Infectious and parasitic dermatoses

Given the high correlation with histopathological features, non‐invasive imaging shows also promising results in inflammatory and infectious skin diseases.^26^, ^27^

In infectious diseases, such as mycoses and parasitic dermatoses, RCM is suitable for direct pathogen diagnosis. Especially in superficial mycoses, such as tinea corporis, tinea cruris, and tinea manuum, and even in tinea incognita, which is difficult to differentiate clinically, direct detection of mycotic material in the epidermis is often achieved by RCM.^28^, ^29^, ^30^, ^31^ Apart from Trichophyton infections, Candida pseudofilaments and conidia^26^ and Malassezia species with typical “spaghetti and meatball” equivalents have been detected by RCM in affected regions.^27^

In case of onychomycosis, good visualization of mycelium and/or spores is ensured even in deeper nail regions, given that the optical character of the nail allows for a higher penetration depth. Hyphae and spores present as brightly reflective structures with typical morphology.^29^, ^32^ Accordingly, RCM achieves a high predictive value and a high specificity in diagnosis of onychomycosis^33^ and has even shown superiority to many conventional detection methods in comparative studies.^29^, ^32^, ^34^

Parasitic mites, such as *Sarcoptes scabiei* or *Demodex folliculorum*, can also be clearly identified. For example, RCM enables the rapid diagnosis of scabies with high sensitivity and specificity.^35^, ^36^, ^37^, ^38^ With respect to therapy monitoring of rosacea, RCM permits quantification of mite density that is superior to histology^38^, ^39^ and is also suitable for the diagnosis of other Demodex‐related diseases, such as Demodex blepharitis.^40^

#### Inflammatory dermatoses

Psoriasiform dermatitis is strongly associated with thickening of the epidermis, whereas spongiotic dermatitis has the typical RCM features of epidermal spongiosis and vesicle formation. Prototypic diseases of psoriasiform dermatitis, such as plaque psoriasis and seborrheic dermatitis, can be further distinguished based on their morphological differences.^41^, ^42^ However, larger studies on sensitivity and specificity are currently not yet available.

Typical examples of spongiotic dermatitis include irritant and allergic contact dermatitis. Both can be differentiated from each other by RCM – by assessing the reaction of the stratum corneum, the presence of epithelial necroses, and different kinetics.^43^, ^44^, ^45^ Unclear patch‐test results can thus be reliably classified experimentally, without the need for biopsies.^43^, ^46^, ^47^, ^48^

### Limitations

In general, RCM requires detailed knowledge of skin histology and pathology for the correct interpretation of images. Therefore, this technique should be learned in training courses. The greatest limitation of RCM is the shallow penetration depth into the papillary layer of the dermis. Accordingly, all deeper dermal changes, for example in nodular melanomas, nodular basal cell carcinomas, or panniculitis, are missed in confocal diagnostics. It is only suitable for the diagnosis of diseases and tumors with characteristic changes in the epidermis and upper dermis. The relatively long measurement time and the small measuring field may also be limiting factors. Patients must be able to keep still for several minutes. Strongly protruding, sunken, keratotic, or weeping skin lesions are difficult to measure, given that surface changes may result in artifacts and shadowing effects.

## *Ex vivo* REFLECTANCE CONFOCAL MICROSCOPY (*Ex vivo* RCM)

### Technique

*Ex vivo* reflectance confocal microscopy (*ex vivo* RCM) is particularly well suited for examining freshly excised tissue in Moh's surgery margin assessment^15^ and has fewer limitations in terms of penetration depth, given that the samples are mounted with the respective cutting surface.^49^

*Ex vivo* RCM allows for histological examination of the skin within minutes without impairing subsequent, conventional histological examination. After fluorescence staining, the samples are examined by laser light analogous to in vivo RCM,^51^ and images are generated using a reflection mode (RM) and/or fluorescence mode (FM).^52^

Apart from the different reflective indices of cellular compartments,^53^ the fluorescence can be used to create additional contrast that is determined by the histological structure and the properties of the used fluorophore.

While numerous fluorescent dyes have been described,^54^ acridine orange has proven its worth in practice due to its good contrast between nucleus and cytoplasm and its minor bleaching.^50^, ^55^ Acquired images can be converted by the software into images mimicking hematoxylin and eosin (H&E) staining.^56^

### Spectrum of indications

#### Basal cell carcinoma

*Ex vivo* RCM is suited particularly well for the histological diagnosis of basal cell carcinoma. In digital staining, tumor cell proliferates present with identical morphology compared to classical H&E staining (Figure 2).^57^ This can be used both for confirmation by biopsy and in Moh's surgery.

Due to the short time required to obtain the histological image, *ex vivo* RCM presents an alternative to conventional (cryostat‐based) Moh's surgery margin assessment. Moreover, in contrast to classical Moh's surgery, *ex vivo* RCM does not result in tissue loss. In several studies, sensitivity and specificity of the method have been reported as 73%–100% and 90%, respectively.^58^, ^59^, ^60^, ^61^

#### Squamous cell carcinoma, Bowen's disease, and actinic keratosis

The visualization of cutaneous squamous cell carcinomas by *ex vivo* RCM with digital staining is similar to conventional H&E sections; however, tumor cells are less eosinophilic while hyperkeratosis results in stronger reflection. Nuclear staining with acridine orange permits detection of nuclear pleomorphisms and atypical mitotic figures. An invasive growth pattern is clearly identified.^55^ Given the detailed visualization of nuclear pleomorphisms and mitotic figures, the morphology of actinic keratosis and Bowen's disease is identical to H&E sections, and these entities are well differentiated from squamous cell carcinoma.^62^

#### Melanocytic tumors

The parameters for classification of melanocytic tumors were defined already in 2017, analogous to in vivo RCM.^53^ Overall, the assessment appears to be difficult due to the strong reflection of melanin. Therefore, while non‐pigmented melanocytic nevi can certainly be identified as such,^49^ the technique is not (yet) suitable for the reliable differentiation of melanomas and nevi.

#### Inflammatory dermatoses

Through application of *pattern analysis* according to Ackermann,^63^ *ex vivo* RCM enables an initial assessment of inflammatory dermatoses within a few minutes. For this purpose, the arrangement of the inflammatory cellular infiltrate in the dermis (superficial, deep, perivascular, perifollicular, interstitial, lichenoid) or in adipose tissue (septal versus lobular panniculitis) is assessed.^64^

Additional features of inflammatory dermatoses, such as spongiosis or acanthosis, are also clearly identified.^64^ In blistering dermatoses, intraepidermal and subepidermal blister formation can be distinguished. In terms of perspective, the technique may also be used for direct immunofluorescence analysis on wet mounts.^65^ The examined biopsies can subsequently be processed without tissue loss for conventional histology and immunohistochemistry.^63^

### Limitations

In addition to the optimization of sample preparation, staining, and technical image generation, current challenges include, in particular, the correct interpretation of the microscopic *ex vivo* image.^66^

Imaging of large tissue samples may be challenging, given that the freshly excised, unfixed tissue sample should not be thicker than a few millimeters and often requires macroscopic manual trimming.^67^

Due to variations in sample thickness, density, and quality, it is also necessary to adjust the pressure applied during tissue mounting as evenly as possible, to ensure a level tissue surface.^49^, ^51^

Irregularities in tissue surface caused by, for example, sample contamination, incomplete contact to the slide due to air bubbles, and insufficient ultrasound gel as medium for the water immersion lens, may result in the artifacts described above and limit image interpretation.^68^

For tissue portions that are not visualized, serial imaging at different penetration depths followed by fusion can minimize artifacts and facilitate automatic pattern recognition using artificial intelligence (AI).^69^

Given that blurry staining is often observed when analyzing frozen sections, *ex vivo* RCM on fresh tissue is recommended.^70^

Limitations of the utilization of AI in *ex vivo* RCM include technical challenges such as insufficient tumor identification by AI in cases of unclear tumor borders, and unequal or insufficient contrast, especially in cases of coinciding dense inflammatory infiltrates. In addition, increased error rates are observed in the differentiation of tumor portions and skin appendages. In *ex vivo* RCM, suboptimal flattening of the tissue prior to scanning results in many artifacts and will, therefore, impede the interpretation not only for experts but also for AI.^71^

## OPTICAL COHERENCE TOMOGRAPHY (OCT)

### Technique

In optical coherence tomography (OCT), light beams are sent into the tissue, usually with a superluminescent diode,^72^ and the differences in transit time of the reflected light portions are recorded. The coherence length of the light source defines the axial resolution of 3–15 µm and the lens optics defines the lateral resolution of up to 15 µm.^73^ Wavelength and, indirectly, light scattering by the skin limit the penetration depth into the skin to 1–2 mm. After amplification of the signal intensity, vertical 2D‐images can be generated based on a logarithmic grayscale or false color scale.^73^, ^74^ Structural or conventional OCT images are similar to images of histological sections. In addition, some instruments are equipped with dynamic OCT software. These are also referred to as angiographic or dynamic OCT (D‐OCT). D‐OCT is based on the principle of *speckle‐variance OCT*. This means that the software shows moving particles as red superimposition over the gray‐white structural OCT image.^75^

### Indications

#### Basal cell carcinoma

Apart from its importance in the diagnosis of basal cell carcinoma, OCT allows conclusions to be drawn on the underlying histological subtype by visualization of specific epidermal and dermal morphologies: In vertical and *en face* view of structural OCT imaging, nodular basal cell carcinomas present as a dermal hypo‐reflective ovoid structure with hypo‐reflective clefting and a hypo‐reflective rim (Figure 2).^76^ The nests are always found near hair follicles and are typically associated with the hair shaft.^77^ In the hyper‐reflective compact connective tissue rim of the tumor nests, elongated, oval, and partly branched vessels with a diameter of up to 300 µm are found.^77^ Important criteria of superficial basal carcinomas include hypo‐reflective nests or ovoid structures originating from the epidermis with hypo‐reflective protrusions into the dermis.^76^ The vessels present without branches, thin (< 40 µm), short (< 80 µm) and with a loose helical course in the area of the DEJ.^77^ Typical of sclerodermiform basal cell carcinoma is a racemose presentation with multiple nodular structures separated from the epidermis or smaller aggregating groups of nests.^76^ Typically, the tumor nests are surrounded by dilated vessels.^77^

#### Actinic keratosis and squamous cell carcinoma

OCT may also be a suitable procedure for diagnosis of cutaneous squamous cell carcinoma and in the differentiation of squamous cell carcinoma, actinic keratosis, and Bowen's disease.^78^ Actinic keratoses are characterized by morphological criteria, such as abnormal architecture of the epidermal cellular layers, thickened epidermis, and evidence of epidermal hyper‐reflective morphology consisting of streaks and dots.^79^ If squamous cell carcinoma is suspected, the integrity of the DEJ should be assessed initially. If this cannot be confirmed reliably or completely, invasive infiltration by squamous cell carcinoma should be suspected. This is corroborated if hyper‐reflective epidermal tumor infiltration with blurring of DEJ or epithelial periadnexal infiltration is observed.^80^

#### Melanocytic lesions (nevi and melanomas)

Structural OCT is of minor significance in the diagnosis of melanocytic lesions, given that unambiguous discrimination of nevi and malignant melanomas is impossible due to the insufficient resolution of structural OCT.^81^, ^82^

Another criterium to differentiate between benign and malignant melanocytic lesions is the assessment of the vascular pattern. Welzel et al. could show by means of D‐OCT that melanomas have more blood vessels than the surrounding healthy skin, and that these also exhibit a chaotic vascular pattern.^83^ The features “atypically formed and irregularly distributed vessels, increased vessel density and increased vessel diameter” were significantly associated with high‐risk melanomas and metastatic melanomas.^83^ Moreover, it was shown that atypic vessels are positively correlated with the Breslow index.^84^

#### Inflammatory and infectious skin diseases

Inflammatory and infectious dermatoses associated with changes in epidermis and circulation can be examined by (D‐)OCT. In contrast to the assessment of nonmelanoma skin cancer (NMSC), there is currently little evidence for the examination of most inflammatory and infectious skin diseases, and the indications are rather experimental in nature.

## Limitations

The most important limitation of OCT is the low resolution at the expense of the high penetration depth due to physical restrictions. Given the low resolution, individual cells cannot be visualized. Moreover, structural differentiation of melanocytic lesions is not possible. This limitation has now been overcome by the development of OCT into *line‐field* confocal optical coherence tomography (LC‐OCT). For optimal measurement without motion artifacts, a stable positioning of the patient is required to avoid movement by patient and investigator. Dynamic motion artifacts manifest as horizontal lines. The best results are achieved by stabilizing the OCT instrument during measurement with both hands. No preparation of the skin with gel or oil is required for OCT examination. The measurement is therefore fast, taking approximately 30 seconds without dynamic mode and 60 seconds with D‐OCT.

## *LINE‐FIELD* CONFOCAL OCT (LC‐OCT)

### Technique

*Line‐field* confocal OCT (LC‐OCT) combines the principles of OCT and RCM, thus enabling high‐resolution (1–2 µm) visualization of the skin down to the mid‐dermis (approximately 500 µm). The instrument consists of a two‐beam interference microscope with a continuous laser source with a wavelength of 800 nm and a line scan camera as photodetector.

In detail, LC‐OCT is based on time‐domain OCT (TD‐OCT) generating multiple, parallel A‐scans from the skin surface down to a depth of 500 µm for the acquisition of B‐images while the instrument is constantly refocusing. Dynamic live focusing of B‐scans allows for a high frame rate, lateral (1.3 µm) and axial (1.1 µm) resolution. The images are black‐gray‐white. Their contrast is generated by the varying degrees of reflection of natural chromophores in the skin, such as keratin and melanin. For example, due to the relatively high refractive index compared to air and water, pigmented cells are depicted as very bright, contrary to the dark cytoplasm, which is rich in water. The images are generated in real time in three modes: vertical (*en coupe*) as in OCT and histology, horizontal (*en face*) as in RCM and dermoscopy, and 3D. Video recordings are also possible. Navigation on the instrument is controlled by a dermoscopic camera to facilitate the exact localization of the scan.

Given that the incorporated laser corresponds to laser classification 1M according to EN 60825‐1, it is approved for use without special protective equipment for patients, including children and pregnant women.

### Indications

#### Basal cell carcinoma

LC‐OCT allows for morphological diagnosis and subtyping of basal cell carcinomas: Nodular basal cell carcinomas show atypic keratinocytes, altered DEJ, tumor nests in the dermis, hypo‐reflective clefting, prominent vascularization, and white hyper‐reflective stroma, whereas superficial basal cell carcinomas present with thickening of the epidermis due to tumor nodes with string of pearl pattern, and sclerodermiform basal cell carcinomas with elongated hypo‐reflective tumor strands surrounded by bright collagen (shoal of fish pattern) (Figure 2).^85^, ^86^, ^87^

#### Field cancerization

In numerous studies, various specific stages of keratinocyte tumors have been analyzed by LC‐OCT. The focus was on the morphology of keratinocytes and the architecture of epidermis and DEJ.^88^, ^89^, ^90^ Typical features shown with LC‐OCT include hyperkeratosis/parakeratosis, disruption of stratum corneum, broadened epidermis, basal and suprabasal keratinocyte atypia, dilated vessels, and collagen alterations. While squamous cell carcinomas present with an interrupted dermal‐epidermal junction zone as well as ulceration and keratin plugs, the DEJ is usually clearly identified in actinic keratoses and Bowen's disease. Based on the basal growth pattern of keratinocytes, LC‐OCT is also able to classify actinic keratoses reproducing the histological PRO classification.^91^

#### Melanocytic lesions

LC‐OCT provides the opportunity to assess melanocytic lesions. Similar to RCM, the high resolution enables analysis of individual cells and may thus facilitate differentiation of nevi and melanomas.^92^, ^93^, ^94^, ^95^ Benign nevi show wavelike structures in papillary and reticular dermis corresponding to melanocytic strands/nests (*wave pattern*).^92^ Irregular honeycomb patterns, pagetoid growth of large, round hyper‐refractive cells in the epidermis, and absence of well‐defined, homogenous dermal nests are characteristic for the diagnosis of melanomas (Figure 1). In one study, LC‐OCT showed high sensitivity and specificity for the diagnosis of melanomas, although differentiation from dysplastic nevi is still unclear.^93^

#### Inflammatory dermatoses

Due to its fast cellular resolution, LC‐OCT facilitates visualization of epidermis, DEJ, and dermis to a depth of 500 µm. In inflammatory dermatoses, it may enable correlation with in vivo histopathology. The best‐studied skin diseases include autoimmune bullous dermatoses, contact eczema, and psoriasis, although the studies performed so far should be seen as preliminary. Characteristic features, such as spongiosis and vesicle formation, can be depicted. While inflammatory cells appear as refractile elements, subtyping is usually not possible. Given that the level of split formation can be determined intuitively, however, this can be used successfully for the non‐invasive diagnosis of pemphigus foliaceus, pemphigus vulgaris, and bullous pemphigoid if clinically suspected.^96^ In addition, there have been anecdotal reports for the use in pustular dermatoses.^97^ Plaque psoriasis is characterized by thickening of stratum corneum and epidermis, elongated rete ridges, and hypo‐refractive, elongated dermal papillae in LC‐OCT. Munro's microabscesses in the form of subcorneal conglomerates of hyper‐refractive cells are less common. In contrast, eczemas are predominantly characterized by thickened and disrupted stratum corneum with alternating hypo‐ and hyper‐refractive layers as well as spongiosis and vesicle formation.^98^ Given that systematic studies on these topics are not yet available, diagnostic criteria need to be further defined and standardized.

### Limitations

While LC‐OCT is very intuitive compared to, for example, RCM, the correct interpretation of images requires detailed knowledge of histology and pathology of the skin. Accordingly, this technique should be learned in training courses. The resolution is almost cellular, although nosological classification of individual cells is sometimes difficult. This applies especially to inflammatory cells. Due to the penetration depth to the mid‐dermis, deeper dermal changes – for example, nodular melanomas, nodular basal cell carcinomas, or panniculitis – are not adequately assessed, especially in thicker lesions. Similar to histology or dermoscopy, the method remains user‐dependent.

## MULTIPHOTON TOMOGRAPHY

### Technique

Multiphoton tomography (MPT) is based on the principle of fluorescence excitation of endogenous molecules by two or more photons. In contrast to single photon microscopy where fluorophores are excited by relatively high‐energy, short‐wave radiation, imaging by MPT is based on energy emissions by means of long‐wave radiation in the near infrared spectrum.^99^

Epidermal and dermal structures are visualized in high resolution by autofluorescent molecules, such as keratin, melanin, elastin, porphyrins, as well as free and protein‐bound NADH, and the phenomenon of *second harmonic generation* (SHG). Integrated Fluorescence Lifetime Imaging (FLIM) allows also for the analysis of the cellular metabolic state and molecular finger prints.^100^, ^101^ MPT produces images of horizontal sections, but reconstruction of these images also permits three‐dimensional tissue assessment.^102^

A particular advantage of MPT is the intravital analysis at high resolution without requirement for prior staining or labeling of tissue. In this respect, by eliminating the artifacts induced by these methods, such as shrinking or influx of fluid into the intracellular space, MPT is superior to conventional histology.

### Indications

#### Actinic keratosis

Histopathologic characteristics of actinic keratosis in MPT include acanthosis, pleomorphic keratinocytes, shifted nucleus‐cytoplasm ratio in favor of nuclei, as well as reduced cell density and increased, irregular intracellular spaces.^103^ AK cells present with heterogeneous fluorescence patterns and forms.^103^ In addition, increased collagen content below the AK with surrounding solar elastosis of sun‐exposed skin can be visualized.^103^, ^104^, ^105^

#### Squamous cell carcinoma

In MPT, squamous cell carcinomas present with hyperkeratosis in all epidermal layers.^103^ Fluorescent cell compartments in corneocytes and keratin bundles corresponding to histological keratin pearls of squamous cell carcinoma are also depicted.^106^, ^107^ The microscopic changes of actinic keratosis, such as increased nucleus‐cytoplasm ratio and reduced cell density in the individual epidermal layers, are significantly more pronounced in squamous cell carcinoma.^103^

#### Basal cell carcinoma

In MPT, basal cell carcinoma is characterized by a loss of organization of the epidermal layers and monomorphic, elongated tumor cells and nuclei densely packed and polarized in one or two directions.^108^ At an excitation of 760 nm, nests of tumor cells can be visualized by surrounding collagen and elastin fibers in parallel organization. Tumor cells have a longer fluorescence lifetime than the surrounding matrix.^109^ Given that tumor cells cannot be visualized at a wavelength of 800–820 nm, the extracellular matrix appears surrounded by dark dermal spaces (*phantom islands*).^108^, ^109^

#### Melanoma

Typical histopathological alterations of melanoma, such as immigrated melanocytes in the epidermis and enlarged intracellular spaces with blurry demarcation to melanocytes can be depicted on MPT images.^110^ Moreover, pleomorphic, atypical melanocytes, cellular fragment and dendritic cells with highly fluorescent dendrites are present in all epidermal layers.^110^, ^111^ Dimitrow et al. described the in vivo diagnosis of melanoma by MPT with a sensitivity of 75% and a specificity of 80%.^110^ The differentiation from melanocytic nevi by multiphoton tomography has been examined in several studies. In contrast to melanoma, these are characterized by monomorphic and clearly defined cells and a regular cellular architecture.^112^ No significant difference between melanocytic cells in melanoma and melanocytic nevi has been observed with respect to fluorescence lifetime.^113^

#### Inflammatory dermatoses

##### Atopic dermatitis

MPT can visualize the characteristic histological features of atypical dermatitis, such as thickened epidermis and enlarged intracellular spaces caused by inflammation‐related edemas.^114^ Compared to classical histology, MPT proves its advantage by the absence of artifacts caused by embedding and staining procedures. Apart from these pathological changes, MPT can reveal the reorganization of mitochondria with perinuclear accumulation.^114^, ^115^, ^116^ Determination of the fluorescence lifetime ratio for free and protein‐bound NADH permits the calculation of the so‐called mean fluorescence lifetime corresponding to the cellular metabolic state.^117^, ^118^, ^119^ Huck et al. have demonstrated a reduced tau_m_ in the stratum granulosum of patients with atopic dermatitis correlating with the severity of the disease. In addition, they could demonstrate a reduced tau_m_ in non‐lesional skin of patients with atopic dermatitis whose subclinical inflammatory metabolism remains usually occult in classical histology.^114^

##### Psoriasis vulgaris

Psoriasis vulgaris presents with acanthosis, parakeratosis and Munro's abscesses in MPT.^120^ In addition, a characteristic, punctuated fluorescence pattern is observed below the stratum corneum of psoriasiform plaques, probably caused by the parakeratosis of psoriasis.^121^ Elongated and dilated papillae have been described using MPT.^121^ Zurauskas et al. examined the fluorescence decay times of psoriasis patients with MPT and could demonstrate a correlation with the *Psoriasis Area and Severity Index* (PASI).^122^ This was facilitated by fully automated analysis of optic biopsies.

### Limitations

In general, MPT requires extensive histopathological knowledge. Image interpretation is often impeded by the horizontal presentation of sections. Another limitation is the low penetration depth of approximately 200 µm.^101^ Assessment of severely wrinkled, sunken, protruding, hyperkeratotic, or weeping lesions is limited. Due to the high‐resolution images on the subcellular level, movement artifacts should be minimized.

Another limitation of MPT is the high cost of purchase and associated maintenance. A darkened and temperature‐controlled room is required for measurements. Older MPT systems are limited by their size and the restricted motion of the optical arm. The newer generations of MPT, with a more compact, cooling‐free design and a more mobile arm, are more practical for everyday clinical use.^123^, ^124^

## ADDITIONAL TECHNIQUES

### Optoacoustic techniques

In optoacoustic imaging, ultrashort light impulses in the visible to infrared spectrum are absorbed by respective biomolecules in tissue (for example, melanin, hemoglobin, lipids, protein, water) and generate ultrasound waves by local thermoelastic expansion (photoacoustic effect). Subsequently, algorithms translate the detected sound waves into a three‐dimensional presentation of macroscopic, mesoscopic, or microscopic resolution.^125^, ^126^, ^127^

#### Multispectral optoacoustic tomography (MSOT)

Multispectral optoacoustic tomography (MSOT) has a high penetration depth with good resolution (penetration depth > 10 mm, lateral resolution 100–500 µm, axial resolution 100–500 µm).^128^ MSOT has already provided promising results in various dermatological areas, such as detection of melanoma metastases in sentinel lymph nodes (SLN), size determination of (nonmelanoma) skin cancer, or determination of inflammatory activity in psoriasis arthritis. Instruments with a CE certificate are already commercially available for research purposes.

#### Raster‐scan optoacoustic mesoscopy (RSOM)

Raster‐scan optoacoustic mesoscopy (RSOM) combines the advantages of deeply penetrating MSOT and high‐resolution optoacoustic microscopy (OAM) and provides a particularly good ratio of penetration depth and image resolution (penetration depth 0–10 mm, lateral resolution < 100 µm, axial resolution 10–100 µm).^128^ RSOM has the potential to complement non‐invasive diagnostics and microscopically controlled surgery not only in the field of chronic inflammatory dermatoses, but also in melanocytic neoplasms.^129^, ^130^, ^131^, ^132^

Limiting aspects of RSOM imaging include the long acquisition time, the susceptibility to motion (for example, breathing), and the low penetration depth restricting the image quality at higher depth. Moreover, penetration depth and thus image quality is restricted in skin types V and VI according to Fitzpatrick, due to the strong absorption by melanin. In addition, dimensions and flexibility of the instruments impede measurements in difficult‐to‐reach, uneven sites of the body and in patients with restricted mobility.^126^, ^133^

#### Optoacoustic microscopy

While optoacoustic microscopy provides the highest resolution of the three procedures mentioned, it is associated with a lower penetration depth (penetration depth 1–2 mm, lateral resolution < 50 µm, axial resolution < 30 µm).^128^ So far, OAM has only been used in experimental studies. In other disciplines (for example, ophthalmology, cardiology, gastroenterology, and gynecology), application of OAM has been investigated in preclinical studies.^134^, ^135^, ^136^

### Multispectral analysis

Multispectral analysis acquires tissue reflection data in visible to infrared wavelengths and allows for precise quantification and analysis of spectral, colorimetric, and spatial features of skin components.

As a sensitive, fully automated, non‐invasive, and user‐friendly imaging method, multispectral analysis may present a potential, additional screening instrument. Given the low specificity and user‐dependent image quality, multispectral analysis is currently not used in daily clinical routine.^137^, ^138^, ^139^, ^140^

### Raman spectroscopy

Raman spectroscopy analyzes the inelastic light scattering of molecules and solid objects. Scientifically, the selective, molecular presentation of skin structures or substances is used for the diagnosis of skin lesions (especially melanomas) and the quantitative analysis of dermal water content, photoaging, topical pharmacokinetics, cosmetics, and visualization of tattoo pigments.^141^, ^142^, ^143^, ^144^

Limiting factors for routine clinical and scientific use are the long acquisition time, limited image quality, and complex technology associated with relatively high costs.^144^

### Micro‐electrical impedance spectroscopy (MIS)

Micro‐electrical impedance spectroscopy (MIS) is not an optical procedure, but measures tissue‐specific resistance (impedance). The result is used to calculate an electrical impedance score (EIS 0–10), which has been evaluated in various studies. This score correlates with certain probabilities of malignancy of keratinocytic or melanocytic lesions. For use in nonmelanoma lesions, seborrheic keratoses, in particular, have to be excluded clinically by other means, to prevent an unnecessarily high number of false positive, elevated EIS. If seborrheic keratoses and inflammatory lesions are excluded clinically or by dermoscopy, MIS may present a valuable decision‐making tool in clinical routine.

### Laser‐induced plasma spectroscopy (LIPS)

Laser‐induced plasma spectroscopy (LIPS) is a new procedure for analyzing the potential malignancy of suspicious lesions. Similar to MIS, the results are assessed via a score which may be helpful for further management of the lesion. Here, a nanosecond impulse from a neodymium‐YAG laser is used to generate a microplasma from the lesion, which is then analyzed spectroscopically using AI‐based software. The technique is painless on the skin and non‐invasive. Three measurements are performed per lesion. In other medical and physical areas, LIPS has already been used as a fast and precise tool (hepatic carcinomas, colorectal carcinomas, breast cancer). The plasma changes (among others, calcium, zinc, and copper concentration) correlate with cell proliferation, apoptosis, and differentiation.^145^

## COMPARISON OF METHODS AT A Glance

Table 1 provides an overview of the presented imaging techniques including technical specifications, primary indications, and equipment costs.

**TABLE 1:** Overview of innovative, non‐invasive imaging techniques for dermatological diagnostics.

	Resolution	Penetration depth	Duration of measurement	Image section (horizontal, vertical, 3D)	Image interpretation	Equipment costs	Primary indication (melanocytic, NMSC, inflammatory)
In vivo RCM	1–3 µm	Up to 250 µm	2–5 min	Horizontal	Detailed knowledge of dermatopathology required; training courses	From 70,000–185,000 € (depending on equipment)	Epithelial and predominantly melanocytic tumors with higher resolution
*Ex vivo* RCM	1–3 µm	Up to 250 µm	< 1–5 min (depending on tissue size)	Horizontal and vertical	Detailed knowledge of dermatopathology required	From 249,000 €	Moh's surgery margin assessment of fresh tissue derived from epithelial and melanocytic tumors; other disciplines (e. g., gynecology and urology)
OCT	10–15 µm	Up to 1.5 mm	0.5–1 min	Horizontal and vertical	Detailed knowledge of dermatopathology required	From 85,000 €	Epithelial tumors with higher penetration depth
LC‐OCT	1–3 µm	Up to approx. 500 µm	2 min	Horizontal, vertical, 3D, and video recordings	Detailed knowledge of dermatopathology required; training courses	From 150,000 €	Epithelial and melanocytic tumors with higher resolution
MPT	0.5–2 µm	Up to 200 µm	5–15 min	Horizontal	Detailed knowledge of dermatopathology required; training courses required for interpretation	From 290,000–325,000 € (depending on equipment)	Inflammatory skin diseases (suspected atopic dermatitis and psoriasis), acute and chronic wounds, epithelial/melanocytic tumors
Optoacoustic	Depending on method, < 1 µm to 100–500 µm lateral and axial	More than 10 mm	1–15 min	3D	Detailed knowledge of dermatopathology required; training courses required for interpretation; user‐dependent	MSOT: 350,000–500,000 € RSOM: 130,000–150,000 €	Melanoma metastases in sentinel lymph nodes; size determination of skin tumors; inflammatory activity and monitoring of therapy in inflammatory diseases
Multispectral analysis	20 µm	Up to 2.5 mm	3 min	Horizontal	Computer‐assisted imaging enables classification of melanocytic lesions	Currently, not commercially available.	Additional screening tool (no routine use in clinical practice yet)
Raman spectroscopy	Depending on method, up to a few µm	Depending on wavelength, up to several hundred µm	3–10 min	Raman spectrum	Spectral analysis compared with reference spectra; user‐dependent; training courses required	Currently, not commercially available	Epithelial/melanocytic tumors
***Non‐optical procedures***				***Output mode***			
Micro‐electrical impedance spectroscopy	–	To upper dermis	3–5 min	EIS	Impedance analysis compared to normal tissue	From 6,900 €	Epithelial/melanocytic tumors
Laser‐induced plasma spectroscopy	–	Epidermal lesions	3–5 min	LIPS score	Plasma spectral analysis compared to normal tissue	From 42,000 €	Epithelial/melanocytic tumors

*Abbr*.: In vivo RCM, in vivo reflectance confocal microscopy; *Ex vivo* RCM, *ex vivo* reflectance confocal microscopy; OCT, optical coherence tomography; LC‐OCT, line‐field confocal optical coherence tomography; MPT, multiphoton tomography; 3D, three‐dimensional; EIS, electrical impedance score; LIPS, laser‐induced plasma spectroscopy.

## CONFLICT OF INTEREST STATEMENT

See long version of the guideline at www.awmf.org.
